# The Parting Message

**Published:** 1891-02-28

**Authors:** 


### THE PARTING MESSAGE.

I shall not tread thy battlefield,
Nor see the blazon on thy shield:
Take thou the sword I could not wield,
And leave me, and forget.
Be fairer, braver, more admired;
So win what feeble hearts desired;
Then leave thine arms, when thou art tired,
To some one nobler yet.—Ionica.

